# The use of d-dimer in the diagnosis and risk assessment of intracardiac thrombus among patients with dilated cardiomyopathy

**DOI:** 10.1038/s41598-023-45077-4

**Published:** 2023-10-23

**Authors:** Yuan Huang, Wang-Wei Zhou, Yu-Xin Li, Xiao-Zhen Chen, Chun Gui

**Affiliations:** 1 Department of Cardiology, Jiangbin Hospital of Guangxi Zhuang Autonomous Region, Nanning, Guangxi China; 2 Department of Cardiology, Liuzhou People’s Hospital, Liuzhou, Guangxi China; 3 Department of Cardiology, The First Affiliated Hospital of Guangxi Medical University, No 6, Shuangyong Road, Nanning, 530021 Guangxi China; 4 Guangxi Key Laboratory Base of Precision Medicine in Cardio-Cerebrovascular Diseases Control and Prevention, Nanning, China; 5 Guangxi Clinical Research Center for Cardio-Cerebrovascular Diseases, Nanning, China

**Keywords:** Biomarkers, Cardiovascular diseases

## Abstract

d-dimer is a biomarker of coagulation and fibrinolytic system activation in response to the body's hypercoagulable state. The study aims to investigate the usefulness of d-dimer in diagnosing and assessing the risk of intracardiac thrombus in patients with dilated cardiomyopathy (DCM). Consecutively enrolled in this study were patients with DCM who were admitted to our center for the first time. The diagnostic value was evaluated using the receiver operating characteristic (ROC) curve. Additionally, we used univariate and multivariate logistic regression to investigate the association between d-dimer and intracardiac thrombus. We also performed smooth curve fitting, threshold saturation effect analysis, and subgroup analysis. In total, 534 patients were enrolled in the study, and among them, 65 patients had intracardiac thrombus. Mural thrombus was the predominant type of thrombus, which was mainly located in the left ventricular apex. The optimal cut-off value of d-dimer for the diagnosis of intracardiac thrombus was 484 ng/mL, with a sensitivity and specificity of 0.769 and 0.646, respectively. In both unadjusted and adjusted logistic regression models, a positive association was found between d-dimer and intracardiac thrombus. Curve fitting and threshold effect analysis revealed two inflection points in the relationship between d-dimer and intracardiac thrombus (non-linear test: P = 0.032). When d-dimer was equal to 362 ng/mL, the odds ratio (OR) was 1, and the risk of thrombus gradually increased until it reached 4096 ng/mL, after which the trend no longer increased. Within this range, a twofold increase in d-dimer was associated with a 103.2% increased risk (OR = 2.032; 95% CI 1.293–3.193; P < 0.01). In the subgroup analysis, there was a significant interaction between d-dimer and BMI on intracardiac thrombus (P value for interaction was 0.013), and the risk was higher in patients with a BMI ≥ 25 kg/m^2^ (OR = 3.44; 95% CI 1.86–6.36; P < 0.01).

## Introduction

Dilated cardiomyopathy (DCM) is a condition characterized by left ventricular enlargement and systolic dysfunction in the absence of coronary artery disease or abnormal burden conditions, which is proportional to the degree of left ventricular injury^1^. The prevalence of DCM has been reported to range from 7 to 36.5 per 100,000 individuals, with a male-to-female ratio of approximately 3.4:1^2–9^.

Intracardiac thrombus is a common complication^10^ of DCM resulting from reduced cardiac output, relative blood stasis in the dilated ventricle, poor myocardial contractility, abnormalities of ventricular wall motion, and possible complications of atrial fibrillation^11^. The presence of a left ventricular thrombus can complicate left ventricular systolic dysfunction and increase the risk of thromboembolic complications, leading to a poor prognosis. One study found that the presence of left ventricular thrombus was associated with a high risk of major adverse cardiovascular events (MACE) (37.1%), embolic complications (22.2%), and mortality (18.9%)^12^. d-dimer, a product of fibrin formation and degradation, can be measured in whole blood or plasma and is a reliable biological indicator of hypercoagulability^13^. It is widely used in the diagnosis of venous thromboembolism (VTE)^14,15^, and has also been studied in diseases related to coagulation and fibrinolytic system activation, including diffuse intravascular coagulation^16^, acute aortic dissection^17^, pulmonary embolism^18^, coronary atherosclerosis^19^, acute myocardial infarction^20^, stroke^21^, and aortic aneurysm^22^. Despite its usefulness, few studies have investigated the thrombotic complications of DCM. Therefore, this study aims to analyze the association between d-dimer and intracardiac thrombus in DCM and discuss its value for DCM risk stratification.

## Materials and methods

### Patients

This retrospective study analyzed the cases of patients with DCM who were first admitted to the First Affiliated Hospital of Guangxi Medical University between October 2012 and May 2020. A total of 860 patients were diagnosed with DCM based on the criteria outlined in the scientific statement established by the American Heart Association^23^. Exclusion criteria were defined for the study and included the following: (1) acute pulmonary embolism; (2) acute stroke; (3) acute myocardial infarction; (4) peripheral arterial and venous embolism; (5) disseminated intravascular coagulation; (6) major surgery, severe trauma, or thrombolytic therapy within three months; (7) individuals under the age of 16; (8) pregnant women; (9) patients with malignant tumors; (10) individuals who underwent previous cardiac pacemaker or left ventricular assist device implantation; (11) individuals who underwent previous heart transplantation; (12) individuals who underwent previous valve surgery; (13) those without inpatient echocardiography data available; (14) those without d-dimer data or critical baseline data available; and (15) individuals who received anticoagulation or antiplatelet therapy prior to admission. The study enrolled a total of 534 patients.

### Data collection

A variety of variables were collected and analyzed for the study subjects, including population characteristics, medical history and comorbidities, physical examination, blood biochemistry, CHADS_2_ score^24^, electrocardiogram, and echocardiogram (detailed information can be found in Supplementary Table 1). All patients underwent these examinations within the first two days of hospitalization, and only the results from the initial examination were considered in cases of repeated examinations. Blood samples were collected within 24 h of admission and analyzed at the inspection center of the First Affiliated Hospital of Guangxi Medical University using the BECKMAN ACL-TOP (USA) immunoturbidimetric quantification method, with normal reference values of 0–450 μg/L for d-dimer. Standardized data collection forms were used to extract all data from electronic medical records, and all co-authors independently reviewed the data to ensure its accuracy. Every hospitalized patient underwent a standardized transthoracic echocardiogram examination. Intracavitary multi-plane observations were conducted, and masses with well-defined borders and poor acoustic contrast with the surrounding endocardium were defined as intracardiac thrombus. The accuracy of the results was confirmed by at least two experienced certified sonographers. In cases where the left atrial appendage was inaccessible or if there were unclear images or doubts about the results, further refinement was achieved through transesophageal echocardiography, as well as cardiac magnetic resonance or enhanced CT scans for confirmation. The length of medical history was determined by identifying the time of onset of symptoms associated with DCM. Pulmonary inflammation, including acute and chronic bronchitis, pneumonia, and chronic obstructive pulmonary disease, was also assessed. The estimated glomerular filtration rate (eGFR) was calculated using the CKD-EPI equation^25^. Pulmonary hypertension was defined as a pulmonary artery pressure of ≥ 30 mmHg estimated from the tricuspid regurgitation pressure difference.

### Statistical analysis

Participants were categorized into thrombus and non-thrombus groups based on the presence of intracardiac thrombus. Normality was tested using the Shapiro–Wilk test. Normally distributed continuous variables were presented as mean ± standard deviation (mean ± SD) and compared between groups using the two independent samples t-test. Skewed continuous variables were presented as median (interquartile range) [median (IQR)] and compared using the Mann–Whitney rank sum test (u test). Categorical variables were presented as the number and percentage [n (%)] and compared using the Pearson chi-square test or Fisher's exact probability method. Bar charts were used to compare d-dimer levels between the thrombus and non-thrombus groups.

The diagnostic accuracy of d-dimer in identifying intracardiac thrombus was assessed by calculating the area under the curve (AUC) of the receiver operating characteristic (ROC) curve, and the optimal cutoff value was determined using the Yordon index. The CHADS_2_ score was used to evaluate the risk of DCM combined with intracardiac thrombus to determine its applicability in this patient population. ROC curves were used to compare the CHADS_2_ score and d-dimer.

We analyzed independent correlations between d-dimer and intracardiac thrombus using univariate and multivariate binary logistic regression models. The odds ratio (OR) and its 95% confidence interval (CI) were used to quantify the risk. Potential covariates (variables with P < 0.1 in univariate analysis) underwent covariate screening by entering them one by one in the basic model of the multivariate logistic regression model or removing them one by one in the full model. We identified potential covariates that changed the initial regression coefficient by more than 10%. We selected appropriate variables for adjustment in the final model by combining clinical analyses^26^. In the multiple collinearity analysis, variables with high variance inflation factors were excluded. For the extended logistic regression model, d-dimer's significantly skewed distribution was accounted for by standardizing, log-transforming, truncation value grouping, and tertile grouping. The extended model approach was used to adjust for potential confounders and evaluate the stability of the relationship between d-dimer and intracardiac thrombus..

Smoothed curve fitting and threshold saturation effect analysis: a generalized additive model was used to assess the nonlinear relationship between serum d-dimer levels and intracardiac thrombus^27^, with corresponding adjustments made in the multivariate-adjusted model^28^. The risk of intracardiac thrombus was described as an OR and 95% CI. The Likelihood Ratio Test was used to perform a nonlinearity test. Inflection points were set based on the smoothing curve, and segmented regression models were constructed accordingly. To achieve an approximately normal distribution, d-dimer was log-transformed. Furthermore, symmetric cropping was performed at the 0.5% level to minimize the effect of outliers^26^. Subgroup analysis was conducted by stratifying relevant effect covariates and drawing forest plots. Likelihood ratio tests were used to calculate P values for interactions.

All statistical analyses were performed using R software (http://www.R-project.org); P < 0.05 was considered statistically significant.

### Ethics approval and consent to participate

The study was conducted following the Declaration of Helsinki (as revised in 2013), and approved by the Ethics Committee of the First Affiliated Hospital of Guangxi Medical University. Written informed consent was obtained from the patient himself or his close relatives.

## Results

### Distribution of intracardiac thrombus

65 out of 534 patients with DCM were identified as having intracardiac thrombus in this study. Among the 65 patients with intracardiac thrombus, 64 (98.5%) had mural thrombi, and only one patient (1.5%) had a free thrombus. Of the 65 patients with intracardiac thrombus, 44 (67.7%) had a single thrombus, while 21 (32.3%) had multiple thrombi. Thrombi were found in various locations: left ventricle (50 cases, 76.9%), left atrial appendage (14 cases, 21.5%), right ventricle (6 cases, 9.2%), and left atrium (1 case, 1.5%). The left ventricular apical thrombi accounted for the highest proportion of left ventricular thrombi (35 cases, 70%). The majority of thrombi did not change position during heart contractions, while only a few were mobile and could move their tails. There was only one case of thrombus that was not attached and was in a free state.

### Population characteristics

**Figure 1:**
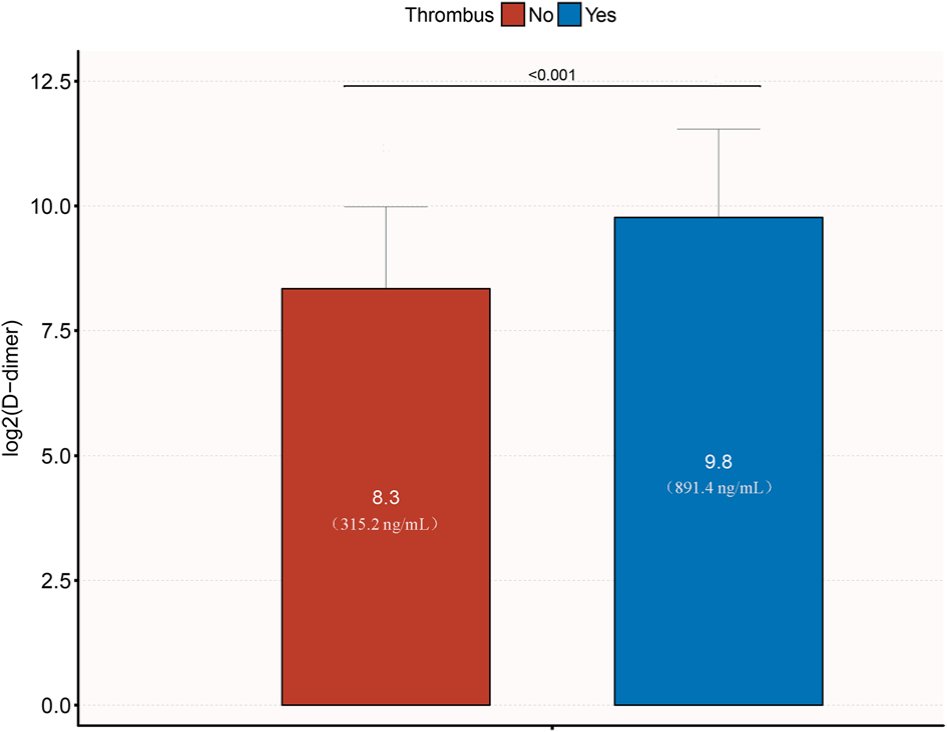
Comparison of plasma d-dimer concentration in thrombus group and non-thrombus group.

Supplementary Table 1 summarizes the population characteristics of the thrombus and non-thrombus groups. Patients in the thrombus group were younger, with a higher proportion of pitting edema in both lower extremities, and had lower systolic pressure and pulse pressure compared to the non-thrombus group (P < 0.05). In terms of biochemical tests, the thrombus group had higher levels of WBC, HB, NE, HCT, RDW-SD, CK-MB, LDH, LD1, α-HBD, d-dimer, PT, INR, AST, ALT, UA, and NT-proBNP, and lower levels of Na^+^, Cl^−^, and ALB (P < 0.05). Echocardiographic results showed lower levels of LVFS and LVEF in the thrombus group (P < 0.05). However, there were no significant differences in comorbidities, electrocardiogram, and CHADS_2_ score between the two groups, as shown in Supplementary Table 1. As depicted in Fig. 1, the plasma d-dimer concentration was significantly higher in the thrombus group (9.8 ± 1.8) than in the non-thrombus group (8.3 ± 1.6) (P < 0.001).

### Accuracy of d-dimer and CHADS_2_ score

**Figure 2:**
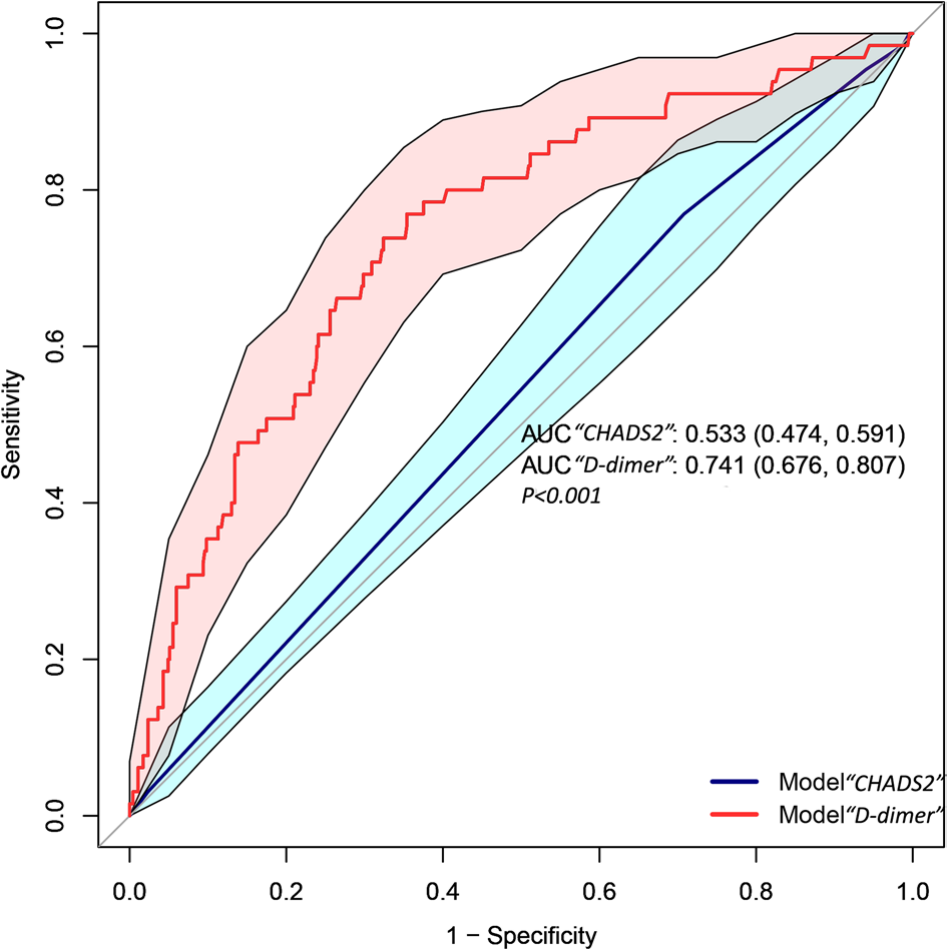
Comparison of the diagnostic accuracy of d-dimer and CHADS_2_ score for intracardiac thrombus. In the figure, the blue and red lines represent their respective ROC curves, and the colored areas represent the corresponding confidence intervals.

The ROC curve in Fig. 2 demonstrated that d-dimer had a higher diagnostic value for DCM combined with intracardiac thrombus, while the CHADS_2_ score had a poorer diagnostic value. The AUC for d-dimer was 0.741 (95% CI 0.676–0.807), with an optimal cut-off value of 484 ng/ml, corresponding to a sensitivity and specificity of 0.769 and 0.646, respectively. Notably, the negative predictive value of d-dimer for the diagnosis of intracardiac thrombus was 0.953, suggesting that a negative d-dimer result could help exclude the diagnosis of intracardiac thrombus (Supplementary Table 2). Conversely, the CHADS_2_ score was not useful in diagnosing combined intracardiac thrombus in patients with DCM, with an AUC of 0.533 (95% CI 0.474–0.591).

### Univariate and multivariate logistic regression analysis

Table 1 displays the unadjusted associations between baseline variables and endpoint events. The results of univariate logistic regression analysis showed that several variables were positively associated with the risk of intracardiac thrombus, including pitting edema in both lower extremities, white blood cell count (WBC), hemoglobin (HB), neutrophil (NE), red cell distribution width-standard deviation (RDW-SD), lactate dehydrogenase (LDH), lactate dehydrogenase 1 (LD1), alpha-hydroxybutyrate dehydrogenase (α-HBD), uric acid (UA), prothrombin time (PT), and international normalized ratio (INR) (P < 0.05). In contrast, age, systolic pressure, pulse pressure, sodium (Na^+^), chloride (Cl^−^), albumin (ALB), left ventricular fractional shortening (LVFS), and left ventricular ejection fraction (LVEF) were negatively associated with the risk of intracardiac thrombus (P < 0.05). Supplementary Table 3 shows that the covariate screening selected variables that had a significant effect on the initial regression coefficients: WBC, LD1, Na^+^, Cl^−^, PT, and ALB. We also included age, gender, smoking history, atrial fibrillation, lung inflammation, chronic obstructive pulmonary disease, diabetes, and pulmonary hypertension in the adjustment based on previous literature and clinical judgment. In addition, we added N-terminal pro-B-type natriuretic peptide (NT-proBNP) and LVEF, which better reflect cardiac function; body mass index (BMI), an important demographic characteristic; and estimated glomerular filtration rate (eGFR), which reflects renal excretory capacity, to the adjustment. Based on these adjustments, we constructed an extended multivariate model.

**Table 1:** Results of univariate logistic regression analysis (*P* < 0.1).

Variables	*OR*(95%*CI*)	*p*-value
Pitting edema in both lower extremities	3.1 (1.71–5.61)	< 0.001
Age	0.97 (0.95–0.98)	< 0.001
Systolic pressure	0.99 (0.97–1)	0.047
Pulse pressure	0.97 (0.95–0.99)	0.003
WBC	1.2 (1.1–1.3)	< 0.001
HB	1.02 (1–1.03)	0.012
NE	1.2 (1.11–1.31)	< 0.001
RDW-SD^Z^	1.31 (1.05–1.65)	0.019
LDH^z^	1.34 (1.04–1.72)	0.023
LD1	1.01 (1–1.01)	0.001
α-HBD	1.01 (1–1.01)	< 0.001
TG	0.64 (0.39–1.06)	0.082
Na^+^	0.93 (0.88–0.98)	0.005
Cl^−^	0.95 (0.91–0.99)	0.024
UA^z^	1.49 (1.15–1.91)	0.002
PT	1.12 (1.05–1.2)	< 0.001
INR	4.91 (2.15–11.19)	< 0.001
d-dimer^z^	1.54 (1.2–1.97)	0.001
ALB	0.93 (0.88–0.98)	0.003
LVFS	0.93 (0.88–0.99)	0.026
LVEF	0.96 (0.93–1)	0.029

ALB: albumin; α-HBD: alpha-hydroxybutyrate dehydrogenase; CI: confidence interval; Cl^−^: serum chloride; HB: hemoglobin; INR: International normalized ratio; LD1: lactate dehydrogenase isoenzyme; LDH: lactate dehydrogenase; LVFS: left ventricular short-axis shortening rate; LVEF: left ventricular ejection fraction; NE: neutrophil count; OR: odds ratio; PT: prothrombin time; RDW-SD: red blood cell distribution width; NA^+^: serum sodium; TG: triglycerides; UA: uric acid; WBC: white blood cell count.

Table 2 shows the unadjusted and adjusted associations between d-dimer and intracardiac thrombus. In the unadjusted model, there was a significant increase in the risk of thrombus as plasma d-dimer levels increased. For each standard deviation increase in d-dimer, the risk of intracardiac thrombus increased by 54% (OR = 1.54; 95% CI 1.2 to 1.97; P < 0.01), while for each twofold increase in d-dimer, the risk increased by 63% (OR = 1.63; 95% CI 1.39 to 1.92; P < 0.01). Compared to the low concentration group (d-dimer < 484 ng/ml), the high concentration group (d-dimer ≥ 484 ng/ml) had an approximately fivefold increased risk of thrombus (OR = 6.08; 95% CI 3.32 to 11.17; P < 0.01). In the tertile grouping comparison, there was a progressive upward trend in the risk of thrombus. The risk of thrombus was significantly higher in T3 than in T1 (OR = 7.78; 95% CI 3.39–17.84; P < 0.01), and the trend test showed a mean 1.96-fold increase in risk for each higher stratum of d-dimer (OR = 2.96; 95% CI 2–4.38; P < 0.01). The extended model, comprising model b adjusted for age, sex, and smoking history, and model C adjusted for age, sex, smoking history, atrial fibrillation, pulmonary inflammation, chronic obstructive pulmonary disease, diabetes, and pulmonary hypertension, did not significantly attenuate the association between d-dimer and intracardiac thrombus. Consequently, model d was further adjusted for NT-proBNP, LVEF, eGFR, BMI, WBC, LD1, Na^+^, Cl^−^, PT, and ALB, which were grouped before inclusion in the model to prevent nonlinear relationships from interfering with the model. LVEF, BMI, and eGFR were grouped with cut-off values of "35%", "25", and "60", respectively, while the remaining variables were converted to categorical variables according to the tertiles. Ultimately, in the full-adjusted model, each standard deviation increase in d-dimer was independently associated with a 40% increase in thrombotic risk (OR = 1.4; 95% CI 1.06–1.85; P < 0.05). In the tertile grouping, there was a significant increase in thrombotic risk in T3 compared with T1 (OR = 4.74; 95% CI 1.84–12.18; P < 0.01).

**Table 2:** Unadjusted and adjusted associations between d-dimer and intracardiac thrombus.

d-dimer (ng/ml)	Total	Event	*OR*^a^ (95%*CI*)	*OR*^b^ (95%*CI*)	*OR*^c^ (95%*CI*)	*OR*^d^ (95%*CI*)
d-dimer^z^	534	65	1.54 (1.2–1.97)*	1.51 (1.17–1.95)*	1.48 (1.14–1.93)*	1.4 (1.06–1.85)^#^
log_2_ (d-dimer)	534	65	1.63 (1.39–1.92)*	1.6 (1.36–1.88)*	1.62 (1.37–1.92)*	1.54 (1.26–1.89)*
d-dimer^G^						
G1 (< 484)	318	15	1(Ref)	1 (Ref)	1(Ref)	1 (Ref)
G2 (≥ 484)	216	50	6.08 (3.32–11.17)*	5.65 (3.06–10.44)*	5.77 (3.09–10.76)*	4.6 (2.31–9.17)*
d-dimer^T^						
T1 (< 225)	178	7	1 (Ref)	1 (Ref)	1 (Ref)	1 (Ref)
T2 (225–605)	178	15	2.25 (0.89–5.65)	2.54 (1–6.46)^#^	2.61 (1.05–6.66)^#^	1.84 (0.6–4.99)
T3 (≥ 605)	178	43	7.78 (3.39–17.84)*	7.42 (3.21–17.14)*	7.7 (3.28–18.08)*	4.74 (1.84–12.18)*
Trend test	534	65	2.96 (2–4.38)*	2.77 (1.88–4.09)*	2.82 (1.9–4.19)*	2.28 (1.46–3.55)*

"*", *P* < 0.01; "#", *P* < 0.05; "Z", Z-Score normalization; "G", group by cutoff value; "T", group by tertile; "*OR*", odds ratio; "*CI*", confidence interval; "Ref", reference. "a": Unadjusted; "b": Adjusted for age, gender, and smoking history; "c": Adjust b and atrial fibrillation, pulmonary inflammation, chronic obstructive pulmonary disease, diabetes, pulmonary hypertension; "d": Adjusted for c and NT-proBNP, LVEF, eGFR, BMI, WBC, LD1, Na^+^, Cl^−^, PT, ALB.

### Smoothing curve fitting and threshold saturation effect analysis

**Figure 3:**
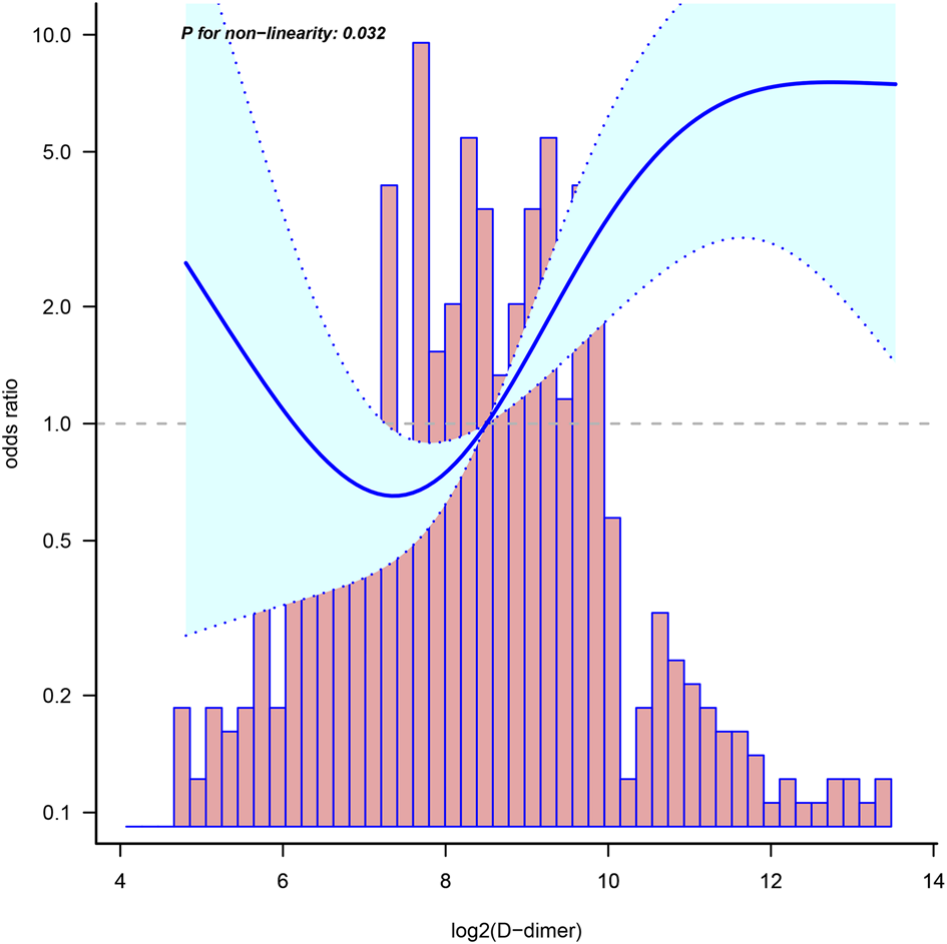
Smooth curve fitting of the relationship between log_2_ (d-dimer) and intracardiac thrombus. The blue solid and dashed lines represent estimates and their corresponding 95% confidence intervals. Only 95% of the data is displayed. Adjusted for all covariates of model d in the expanded model.

We conducted curve fitting analysis to investigate the relationship between d-dimer and intracardiac thrombus, and identified potential inflection points. The fitted curve indicated that there existed a potential nonlinearity in the relationship between d-dimer levels and intracardiac thrombus (non-linear test: P = 0.032). Our analysis revealed that when d-dimer equals log_2_8.5, the odds ratio (OR) for thrombus was 1, and as d-dimer values increased, the risk of thrombus gradually increased until it reached log_2_12. Beyond this point, the trend no longer exhibited an upward trajectory (Fig. 3). Therefore, we established two inflection points (log_2_8.5 and log_2_12) and developed a three-segment linear regression model. Our results indicate that the risk of thrombus increased gradually when d-dimer was between ≥ 362 ng/ml and < 4096 ng/ml, with a 103.2% increase in risk for each twofold increase in d-dimer (OR = 2.032; 95% CI 1.293–3.193; P < 0.01). However, when d-dimer was either < 362 ng/ml or ≥ 4096 ng/ml, we did not observe a statistically significant association (Table 3).

**Table 3:** Threshold effect analysis between log_2_ (d-dimer) and intracardiac thrombus.

	*OR*^a^ (95%CI)	*P*-value
One-line linear regression model	1.54 (1.26–1.89)	< 0.01^#^
Three-stage linear regression model		
d-dimer < 362 ng/ml	1.502 (0.528–4.274)	0.4457
d-dimer ≥ 362 ng/ml, < 4096 ng/ml	2.032 (1.293–3.193)	0.002^#^
d-dimer ≥ 4096 ng/ml	0 (0–infinite)	0.9998
Nonlinearity test	–	0.032^#^

"#", *P* < 0.05; "*OR*", odds ratio; "*CI*", confidence interval; "a": Adjusted for age, gender, smoking history, atrial fibrillation, pulmonary inflammation, chronic obstructive pulmonary disease, diabetes, pulmonary hypertension and NT-proBNP, LVEF, eGFR, BMI, WBC, LD1, Na^+^, Cl^−^, PT, ALB.

### Subgroup analysis and forest plots

**Figure 4:**
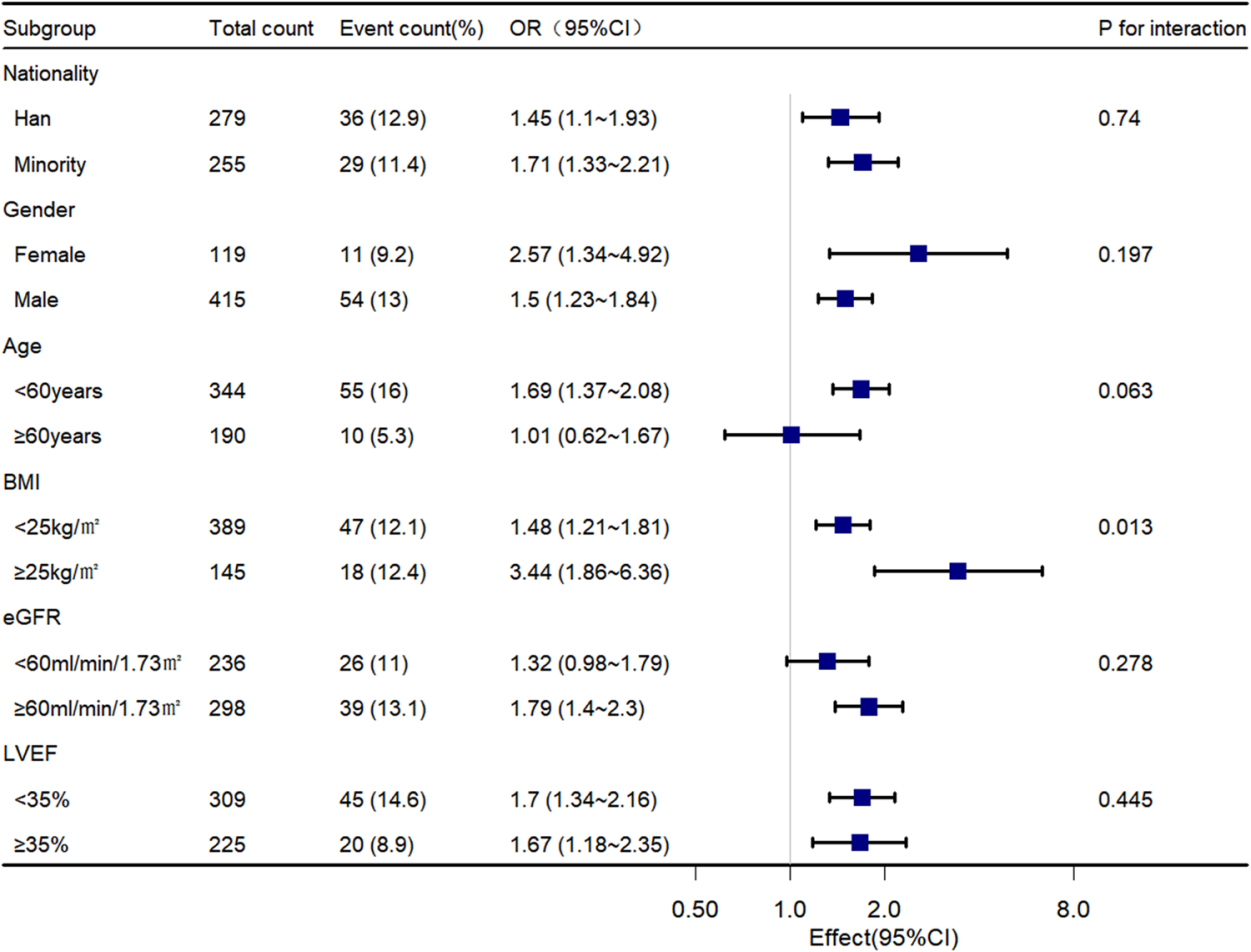
Association of d-dimer with intracardiac thrombus in different subgroups. Each stratification adjusted for all covariates of model d in the expanded model except the stratification factor itself. OR, odds ratio; CI, confidence interval.

To investigate the homogeneity of the relationship between d-dimer and intracardiac thrombus in various subgroups, we performed a subgroup analysis, which we presented using forest plots (Fig. 4). The results revealed that, after adjusting for confounding factors such as age, gender, smoking history, atrial fibrillation, pulmonary inflammation, chronic obstructive pulmonary disease, diabetes, pulmonary hypertension, BMI, NT-proBNP, LVEF, and eGFR, serum d-dimer levels were positively correlated with the risk of intracardiac thrombus across different subgroups. Moreover, we observed a significant interaction between d-dimer and BMI on intracardiac thrombus (P-value for corrected interaction = 0.013), while no interaction was found between d-dimer and nationality (Han, minority), gender (female, male), age (age < 60, age ≥ 60), glomerular filtration rate (eGFR < 60, eGFR ≥ 60), or left ventricular ejection fraction (LVEF < 35%, LVEF ≥ 35%). We found that increased plasma d-dimer was significantly associated with the presence of intracardiac thrombus in subjects with BMI < 25 kg/m^2^, with a 48% increase in risk for each twofold increase in plasma d-dimer (OR = 1.48; 95% CI 1.21 to 1.81; P < 0.01). However, this risk was higher in subjects with BMI ≥ 25 kg/m^2^ (OR = 3.44; 95% CI 1.86–6.36; P < 0.01). Additionally, we observed a higher risk in female subjects at the same d-dimer level than in male subjects. Notably, we did not find a significant association between d-dimer and intracardiac thrombus in the elderly or the subgroup with renal insufficiency.

## Discussion

Dimer is a marker of coagulation and fibrinolytic activation that provides a rapid assessment of thrombotic activity^29–31^. This study found that higher plasma d-dimer levels were associated with an increased risk of intracardiac thrombus. The association was independent of other factors according to an extended logistic regression model. Although the topic of intracardiac thrombus has been of interest, current anticoagulation recommendations for DCM are limited to patients who have already experienced complications of mural thrombus or thromboembolism, as well as patients with combined atrial fibrillation and a CHA_2_DS_2_-VASc score ≥ 2^32^. There is no consensus on anticoagulation for DCM patients with sinus rhythm^33^. Additionally, there is no uniform answer regarding the incidence of intracardiac thrombus in DCM. Previous studies have focused on the incidence of stroke and peripheral vascular embolic events, and the incidence of intracardiac thrombus has been rarely mentioned. Most studies of left ventricular thrombus do not provide a baseline number of DCM^12,34^. In this study, we collected data on DCM patients who were hospitalized for the first time for nearly eight years continuously. We found that the incidence of intracardiac thrombus was about 12.2%. However, this may be overestimated due to stricter inclusion and exclusion criteria. It is important to note that intracardiac thrombus does not equate to stroke or peripheral thromboembolic events. The reported rate of a history of stroke in DCM is low, about 4.5%, and this is not limited to cardioembolic strokes^35^. Moreover, a study reported only 35 thromboembolic events among 159 patients with left ventricular thrombus^12^. This may be related to the characteristics of thrombus in patients with DCM. In this study, we found that most of the thrombi were mural thrombi with poor mobility, making them less likely to fall off. Nevertheless, assessing the risk of thrombosis in DCM is still important since thrombus formation may not only increase the risk of stroke and peripheral thromboembolic events but also exacerbate the progression of heart failure^12^.

The ROC curve analysis indicated that d-dimer has a good diagnostic value for intracardiac thrombus. Previously, we utilized the CHADS_2_ score or the modified CHA_2_DS_2_-VASc^36^ score to evaluate the risk of stroke in nonvalvular AF and select an appropriate anticoagulation regimen. As DCM is a prevalent primary disease of atrial fibrillation and heart failure, it is essential to determine whether these scoring systems can be equally applicable in DCM. However, according to our validation results, the CHADS_2_ score is not appropriate for assessing the risk of intracardiac thrombus in DCM. The likely reasons for this are twofold. Firstly, age, which is a CHADS_2_ risk factor, exhibits a negative correlation in DCM, possibly due to the diverse etiologies of DCM. For instance, myocarditis is a crucial cause of DCM, with biopsy-proven myocarditis progressing to DCM in up to 30% of cases^37^. Myocarditis patients often have severely damaged cardiomyocytes, making them more susceptible to thrombosis^38^. Younger patients are also more likely to develop myocarditis-induced DCM^39^, resulting in this opposite correlation. Secondly, hypertension did not differ significantly in the DCM cohort, whereas lower systolic pressure and pulse pressure were associated with a higher risk of intracardiac thrombus, as per our univariate analysis.

The fitted curve demonstrates that the relationship between d-dimer and intracardiac thrombus forms a sloping "S" shape. The risk of thrombus gradually increases with increasing d-dimer plasma concentration (P < 0.05) between the two inflection points, while the risk threshold is not reached when d-dimer is below 362 ng/mL. Therefore, changes in d-dimer within this interval are not correlated with the risk of thrombus (P > 0.05). However, when d-dimer levels exceed 4096 ng/mL, the risk tends to saturate and no longer rises with the increase of d-dimer. It is important to note that due to the small sample size and large variation range of d-dimer, the confidence interval significantly expanded. Thus, more data is needed to verify the saturation boundary value. Finally, we compared the association between d-dimer and intracardiac thrombus in different subgroups and found that the association was consistent across ethnicity, age, cardiac function, and renal function. Nevertheless, the risk was higher in female and obese (BMI ≥ 25 kg/m^2^) subjects. Several previous studies have confirmed that obesity is a crucial risk factor for arterial and venous thromboembolism, and that obese women have a higher relative risk of deep vein thrombosis than obese men^40,41^. In obese populations, adipose tissue dysfunction may lead to a prothrombotic state, causing reduced fibrinolysis, increased thrombin production, and overactive platelets that directly or indirectly affect hemostasis, coagulation, and fibrinolysis^42^. Additionally, studies have found that obese individuals have elevated levels of fibrinogen^43^, thrombin, and thrombin-antithrombin complex^44^, and that weight loss in morbidly obese patients significantly reduces thrombin generation potential^45^. Furthermore, a large prospective observational single-center cohort study involving 5,000 subjects found obesity to be a key factor in thrombin production^46^. In summary, the interaction between BMI and d-dimer on intracardiac thrombus may be mediated by affecting the coagulation and fibrinolytic processes of the body. Moreover, several studies have reported significantly lower d-dimer levels in men than in women^47–49^, with women being significant predictors of d-dimer positivity^50^. Lastly, it is important to note that due to the inclusion of a population with an average age around the menopausal period, hormonal fluctuations may have significant implications on these findings. Therefore, further investigation is warranted in future studies.

In this study, we investigated the application of d-dimer as an indicator of coagulation and fibrinolysis in DCM complicated with intracardiac thrombus. Elevated d-dimer levels indicate a hypercoagulable state of the body, which is a warning sign for the risk of thrombus, providing more information than echocardiography alone. Our findings also highlight the interaction between d-dimer and thrombus in different subgroups of BMI, emphasizing the need for extra attention to the risk assessment of obese DCM patients. To minimize the influence of potential confounding factors, we used a rigorous statistical adjustment method. Nevertheless, there are some limitations of the study: first, our study may be subject to selection bias because it is a single-center, retrospective study, and despite our efforts to include all eligible patients, some patients were excluded from the study due to lack of d-dimer data at admission. Second, the relatively small sample size and a low number of endpoint events observed after stratification somewhat limited the statistical power of this exploratory study. However, after analysis through multiple levels, perspectives, and models, we found that the association between d-dimer and endpoint events remained robust, and therefore, we consider the conclusions of this study to be reliable. Finally, we could not specify the period of thrombus, nor could we determine how the cardiovascular medications used before admission affected the thrombus status and the d-dimer; and the half-life of d-dimer is only 8 h^51^, so some of the old thrombi may underestimate the risk value of d-dimer. It has been noted^52^ that cardiac magnetic resonance features can distinguish between acute phase thrombus and old thrombus: acute phase thrombus shows high signal intensity on T1- and T2-weighted images, whereas old thrombus shows low signal intensity in T1 and T2 sequences and occasionally shows evidence of calcification. Unfortunately, we lack complete data for this section. In addition, dynamic measurements of d-dimer may be more informative.

## Conclusions

This study explored the optimal cutoff value of d-dimer for diagnosing intracardiac thrombi, depicted the nonlinear relationship between d-dimer and intracardiac thrombi, and conducted stratified analyses in different subgroups. The study provides valuable reference for a better understanding and application of d-dimer in assisting the diagnosis, screening, and risk assessment of intracardiac thrombi in clinical practice. It offers clues for early thrombosis prevention and timely intervention, underscoring its significant clinical utility.

### Supplementary Information

Supplementary Information.

## Data Availability

The datasets used and/or analyzed during the current study are available from the corresponding author on reasonable request.

## Abbreviations

ALB: Albumin
ALT: Alanine aminotransferase
α-HBD: Alpha-hydroxybutyrate dehydrogenase
AST: Aspartate aminotransferase
AUC: Area under the curve
BMI: Body mass index
CK-MB: Creatine kinase isoenzyme
DCM: Dilated cardiomyopathy
eGFR: Estimated glomerular filtration rate
HB: Hemoglobin
HCT: Hematocrit
INR: International normalized ratio
LD1: Lactate dehydrogenase isoenzyme
LDH: Lactate dehydrogenase
LVEF: Left ventricular ejection fraction
LVFS: Left ventricular short-axis shortening rate
NT-proBNP: N terminal pro B type natriuretic peptide
PT: Prothrombin time
RDW-SD: Red blood cell distribution width
ROC: Receiver operating characteristics
CL^−^: Serum chloride
NA^+^: Serum sodium
UA: Uric acid
WBC: White blood cell count
