# Supplementary material for: The use of d-dimer in the diagnosis and risk assessment of intracardiac thrombus among patients with dilated cardiomyopathy
Source: Sci Rep. 2023 Oct 23;13:18075. doi: 10.1038/s41598-023-45077-4 (PMC10593857; doi:10.1038/s41598-023-45077-4)

Supplementary Table 1. Differences between demographic and clinical characteristics of the thrombus and non-thrombus groups.

	**Total (n = 534)**	**Non-thrombus group (n = 469)**	**Thrombus group (n = 65)**	***P*-value**	**Statistic**
**Population characteristics**					
Age(years)	54.5 (45.0, 63.0)	55.0 (46.0, 64.0)	48.0 (36.0, 55.0)	< 0.001	15.248
Gender, n (%)				0.342	0.901
Female	119 (22.3)	108 (23)	11 (16.9)		
Male	415 (77.7)	361 (77)	54 (83.1)		
Nationality, n (%)				0.683	0.166
Han	279 (52.2)	243 (51.8)	36 (55.4)		
Minority	255 (47.8)	226 (48.2)	29 (44.6)		
**Medical history and comorbidities**					
Medical history, n (%)				0.532	1.262
<1 year	259 (48.5)	225 (48)	34 (52.3)		
1~5 years	174 (32.6)	152 (32.4)	22 (33.8)		
≥5 years	101 (18.9)	92 (19.6)	9 (13.8)		
Grade of heart failure (NYHA), n (%)				0.15	Fisher
I	17 (3.2)	15 (3.2)	2 (3.1)		
II	97 (18.2)	91 (19.4)	6 (9.2)		
III	201 (37.6)	177 (37.7)	24 (36.9)		
IV	219 (41.0)	186 (39.7)	33 (50.8)		
Disease status (critical), n (%)				< 0.001	15.774
No	197 (36.9)	188 (40.1)	9 (13.8)		
Yes	337 (63.1)	281 (59.9)	56 (86.2)		
Drinking history, n (%)				0.418	0.655
No	283 (53.0)	245 (52.2)	38 (58.5)		
Yes	251 (47.0)	224 (47.8)	27 (41.5)		
Smoking history, n (%)				0.811	0.057
No	276 (51.7)	241 (51.4)	35 (53.8)		
Yes	258 (48.3)	228 (48.6)	30 (46.2)		
Current smoker, n (%)				0.387	0.748
No	405 (75.8)	359 (76.5)	46 (70.8)		
Yes	129 (24.2)	110 (23.5)	19 (29.2)		
Previous Stroke/TIA, n (%)				0.703	Fisher
No	519 (97.2)	456 (97.2)	63 (96.9)		
Yes	15 (2.8)	13 (2.8)	2 (3.1)		

Pulmonary hypertension, n (%)				0.597	0.279
No	184 (34.5)	164 (35)	20 (30.8)		
Yes	350 (65.5)	305 (65)	45 (69.2)		
Hypertension, n (%)				0.597	0.279
No	435 (81.5)	380 (81)	55 (84.6)		
Yes	99 (18.5)	89 (19)	10 (15.4)		
Atrial fibrillation, n (%)				0.911	0.012
No	434 (81.3)	382 (81.4)	52 (80)		
Yes	100 (18.7)	87 (18.6)	13 (20)		
Diabetes, n (%)				0.763	0.091
No	492 (92.1)	431 (91.9)	61 (93.8)		
Yes	42 (7.9)	38 (8.1)	4 (6.2)		
Respiratory inflammation, n (%)				0.508	0.438
No	320 (59.9)	284 (60.6)	36 (55.4)		
Yes	214 (40.1)	185 (39.4)	29 (44.6)		
COPD, n (%)				1	Fisher
No	518 (97.0)	455 (97)	63 (96.9)		
Yes	16 (3.0)	14 (3)	2 (3.1)		
**Physical examination**					
Pitting edema in both lower extremities, n (%)				< 0.001	14.122
No	252 (47.2)	236 (50.3)	16 (24.6)		
Yes	282 (52.8)	233 (49.7)	49 (75.4)		
Body mass index(㎏/㎡)	23.0 (20.4, 25.2)	23.0 (20.4, 25.2)	22.9 (20.9, 25.2)	0.717	0.132
Systolic pressure(mmHg)	112.0 (102.0, 128.0)	114.0 (102.0, 129.0)	110.0 (103.0, 120.0)	0.049	3.886
Diastolic pressure(mmHg)	75.0 (66.0, 86.0)	75.0 (66.0, 86.0)	76.0 (68.0, 87.0)	0.616	0.252
Pulse pressure(mmHg)	37.0 (30.0, 49.0)	38.0 (30.0, 50.0)	33.0 (27.0, 39.0)	0.001	10.798
Heart rate(times/min)	89.4 ± 18.9	89.2 ± 18.7	90.9 ± 20.1	0.514	0.427
**Blood biochemical**					
White blood cell count($\times10^9$/L)	7.4 (6.2, 9.1)	7.3 (6.1, 8.8)	8.1 (7.2, 10.4)	< 0.001	11.858
Red blood cell count($\times10^{12}$/L)	4.7 (4.3, 5.1)	4.7 (4.3, 5.1)	4.9 (4.5, 5.2)	0.106	2.612
Hemoglobin(g/L)	136.0 (125.0, 147.0)	135.0 (125.0, 147.0)	141.0 (129.0, 155.0)	0.007	7.339
Platelet($\times10^9$/L)	198.5 (164.0, 250.0)	201.0 (166.0, 251.0)	186.0 (154.0, 222.0)	0.084	2.982
Neutrophils($\times10^9$/L)	4.6 (3.6, 5.9)	4.5 (3.5, 5.8)	5.2 (4.2, 7.6)	< 0.001	12.18
Lymphocyte($\times10^9$/L)	1.8 (1.3, 2.3)	1.8 (1.3, 2.2)	1.8 (1.4, 2.3)	0.400	0.707
Neutrophil to Lymphocyte Ratio	2.6 (1.8, 4.0)	2.5 (1.8, 3.8)	3.3 (1.8, 4.8)	0.062	3.484
Hematocrit	0.4 (0.4, 0.5)	0.4 (0.4, 0.5)	0.4 (0.4, 0.5)	0.046	3.971
Platelet distribution width(fl)	0.2 (0.2, 0.2)	0.2 (0.2, 0.2)	0.2 (0.2, 0.2)	0.135	2.23

Red blood cell distribution width-SD	0.15 (0.14, 0.16)	0.15 (0.14, 0.16)	0.16 (0.14, 0.17)	0.006	7.45
Creatine kinase(U/L)	91.0 (61.2, 147.0)	88.0 (61.0, 146.0)	107.0 (70.0, 151.0)	0.166	1.914
Creatine kinase-MB(U/L)	15.0 (11.0, 20.0)	15.0 (11.0, 20.0)	18.0 (11.0, 25.0)	0.020	5.424
Lactic dehydrogenase(U/L)	256.5 (206.0, 318.0)	250.0 (203.0, 309.0)	297.0 (251.0, 385.0)	< 0.001	22.343
Lactic dehydrogenase-1(U/L)	71.0 (55.0, 93.8)	69.0 (54.0, 89.0)	87.0 (72.0, 113.0)	< 0.001	22.697
α-HBD(U/L)	188.0 (148.0, 227.8)	182.0 (145.0, 220.0)	212.0 (181.0, 274.0)	< 0.001	25.526
Total cholesterol(mmol/L)	4.2 (3.5, 4.9)	4.2 (3.5, 4.9)	3.9 (3.4, 4.8)	0.350	0.872
Triglycerides(mmol/L)	1.1 (0.8, 1.4)	1.1 (0.8, 1.4)	1.0 (0.9, 1.3)	0.349	0.878
High-density lipoprotein(mmol/L)	1.0 (0.8, 1.2)	1.0 (0.8, 1.2)	0.9 (0.7, 1.1)	0.112	2.532
Low-density lipoprotein(mmol/L)	2.6 (2.1, 3.2)	2.6 (2.0, 3.2)	2.6 (2.1, 3.1)	0.834	0.044
Homocysteine(μmol/L)	15.2 (12.0, 18.1)	15.2 (11.9, 18.0)	15.6 (12.7, 19.2)	0.233	1.424
Serum potassium(mmol/L)	4.0 (3.7, 4.3)	4.0 (3.7, 4.3)	4.1 (3.7, 4.5)	0.284	1.148
Serum sodium(mmol/L)	139.0 (136.0, 141.0)	139.0 (137.0, 142.0)	137.0 (134.0, 140.0)	0.002	9.196
Serum chlorine(mmol/L)	102.0 (99.0, 106.0)	103.0 (99.0, 106.0)	102.0 (98.0, 104.0)	0.037	4.362
Fibrinogen(g/L)	3.8 (3.0, 4.0)	3.8 (3.0, 4.0)	3.5 (2.6, 4.4)	0.533	0.389
D-dimer(ng/ml)	360.0 (169.5, 755.8)	331.0 (161.0, 655.0)	864.0 (490.0, 1828.0)	< 0.001	39.873
Prothrombin time(s)	13.1 (11.5, 13.8)	12.9 (11.3, 13.4)	13.5 (12.6, 17.0)	< 0.001	18.449
International normalized ratio	1.1 (1.0, 1.2)	1.1 (1.0, 1.1)	1.1 (1.1, 1.4)	< 0.001	18.116
APTT (s)	32.0 (30.1, 33.7)	32.0 (30.3, 33.6)	32.0 (29.6, 34.4)	0.898	0.016
Thrombin time(s)	12.0 (11.3, 12.5)	12.0 (11.3, 12.5)	12.0 (11.5, 12.8)	0.508	0.438
Albumin(g/L)	38.3 (35.4, 41.4)	38.6 (35.5, 41.6)	37.4 (33.6, 39.3)	0.007	7.174
Globulin(g/L)	26.2 (23.0, 29.9)	26.2 (23.2, 30.0)	24.9 (22.6, 28.2)	0.179	1.809
Albumin to Globulin Ratio	1.5 (1.3, 1.7)	1.5 (1.3, 1.7)	1.4 (1.2, 1.7)	0.392	0.732
Aspartate aminotransferase(U/L)	32.0 (23.0, 47.0)	31.0 (23.0, 44.0)	42.0 (30.0, 53.0)	< 0.001	13.861
Alanine aminotransferase(U/L)	31.0 (19.0, 53.8)	30.0 (18.0, 51.0)	39.0 (27.0, 81.0)	< 0.001	13.563
Urea(mmol/L)	7.0 (5.4, 8.9)	7.0 (5.4, 8.9)	7.1 (5.8, 9.1)	0.487	0.483
Creatinine(μmol/L)	93.0 (79.0, 113.0)	93.0 (78.0, 112.0)	99.0 (81.0, 117.0)	0.180	1.8
Cystatin C(mg/L)	1.1 (0.8, 1.3)	1.1 (0.8, 1.3)	1.1 (0.8, 1.4)	0.670	0.181
EGFR(ml/min/1.73m^2)	64.8 (50.2, 85.8)	64.4 (50.0, 85.8)	68.9 (52.4, 85.8)	0.593	0.286
Uric acid(μmol/L)	492.0 (391.2, 629.0)	483.0 (389.0, 609.0)	604.0 (402.0, 702.0)	0.006	7.539
NT-proBNP(pg/ml)	3876.0 (1950.0, 8243.0)	3584.0 (1724.0, 7517.0)	6145.0 (3334.0, 9393.0)	< 0.001	11.633
**Electrocardiogram**					
Ventricular tachycardia/fibrillation, n (%)				1	0
No	485 (90.8)	426 (90.8)	59 (90.8)		
Yes	49 (9.2)	43 (9.2)	6 (9.2)		
**Echocardiographic**					

LAD(mm)	45.5 (41.0, 51.0)	46.0 (41.0, 50.0)	45.0 (41.0, 52.0)	0.916	0.011
LVDd(mm)	69.0 (64.0, 75.0)	69.0 (64.0, 75.0)	70.0 (63.0, 76.0)	0.903	0.015
LVDS(mm)	58.0 (53.0, 63.0)	58.0 (53.0, 63.0)	58.0 (54.0, 64.0)	0.428	0.628
LVFS (%)	16.0 (13.0, 19.0)	16.0 (13.0, 19.0)	15.0 (11.0, 18.0)	0.019	5.464
LVEF (%)	33.0 (26.2, 39.0)	33.0 (27.0, 39.0)	30.0 (24.0, 37.0)	0.027	4.909
Stroke volume(ml/B)	80.5 (62.0, 99.0)	81.0 (62.0, 99.0)	80.0 (54.0, 98.0)	0.324	0.974
Cardiac output(L/min)	7.1 (5.3, 8.9)	7.1 (5.3, 8.9)	7.2 (4.7, 9.2)	0.989	0
$CHADS_2$ score, n (%)				0.754	Fisher
0	13 (2.4)	11 (2.3)	2 (3.1)		
1	369 (69.1)	321 (68.4)	48 (73.8)		
2	121 (22.7)	109 (23.2)	12 (18.5)		
3	24 (4.5)	22 (4.7)	2 (3.1)		
4	5 (0.9)	4 (0.9)	1 (1.5)		
5	2 (0.4)	2 (0.4)	0 (0)		

Abbreviations: APTT, Activated partial thromboplastin time; α-HBD, Alpha-hydroxybutyrate dehydrogenase; COPD, Chronic obstructive pulmonary disease; EGFR, Estimated glomerular filtration rate; LAD, Left atrium anteroposterior dimension; LVDd, Left ventricular end diastolic dimension; LVFS, Left ventricular fractional shortening; LVEF, Left ventricular ejection fraction; NT-proBNP, N terminal pro B type natriuretic peptide; NYHA, New York Heart Association; TIA, Transient ischemic attack.

Supplementary Table 2. Comparison of the diagnostic value of D-dimer and $CHADS_2$ score in the diagnosis of intracardiac thrombus

Variable	D-dimer	$CHADS_2$ score
AUC (95%CI)	74.15% (67.59% ~ 80.70%)	53.26% (47.41% ~ 59.10%)
Sensitivity	0.769	0.769
specificity	0.646	0.292
positive predictive value	0.231	0.131
negative predictive value	0.953	0.901
true negatives	303	137
true positives	50	50
number of false negatives	15	15
number of false positives	166	332
Accuracy	0.661	0.35
precision	0.231	0.131

Abbreviations: AUC (95% CI), area under the receiver operating curve and its 95% confidence interval.

Supplementary Table 3. Covariate Screening.

**Term1**	**Change.percentage1**	**Change.percentage2**	**VIF**	**Colinearity**	**Select**
Crude	Ref.	Ref.	2.718	0	Ref.
pitting edema in both lower extremities	-5.8	-10.6	1.225	0	Yes
Age	-6.5	2.3	1.224	0	No
Systolic pressure	-4.6	-0.1	1.799	0	No
Pulse pressure	-7.6	1.1	1.727	0	No
White blood cell count	-13.1	0	14.877	1	Yes
Hemoglobin	3.4	-5.6	1.344	0	No
Neutrophils	-18.9	-1.9	15.012	1	Yes
Lactic dehydrogenase-1	-10.6	0.3	6.246	1	Yes
α-HBD	-29.9	-9.1	10.845	1	Yes
Triglycerides	-1.6	0.4	1.099	0	No
Serum sodium	-12.7	-0.3	2.563	0	Yes
Serum chlorine	-10.8	-5.6	2.563	0	Yes
Prothrombin time	-31.2	-17.3	404.695	1	Yes
INR	-33.6	-17.2	405.911	1	Yes
Albumin	-15.3	5.1	1.414	0	Yes
LVFS	1.4	0	68.561	1	No
LVEF	1.4	-0.1	68.763	1	No
RDW-SD^Z	-7.3	0	1.365	0	No
Lactic dehydrogenase^z	-3.2	-59.8	7.91	1	Yes
Uric acid^z	-4.1	-4.4	1.203	0	No
**Term2**	**Change.percentage1**	**Change.percentage2**	**VIF**	**Colinearity**	**Select**
Crude	Ref.	Ref.	1.265	0	Ref.
pitting edema in both lower extremities	-5.8	-3.3	1.134	0	No
Age	-6.5	4.3	1.137	0	No
Systolic pressure	-4.6	0	1.802	0	No
Pulse pressure	-7.6	1.6	1.696	0	No
White blood cell count	-13.1	7	1.212	0	**Yes**
Hemoglobin	3.4	-4.1	1.242	0	No
Lactic dehydrogenase-1	-10.6	1.3	1.091	0	**Yes**
Triglycerides	-1.6	-1.9	1.073	0	No
Serum sodium	-12.7	-7.6	2.645	0	**Yes**
Serum chlorine	-10.8	-21.9	2.8	0	**Yes**
Prothrombin time	-31.2	18.8	1.337	0	**Yes**
Albumin	-15.3	15.5	1.296	0	**Yes**
LVFS	1.4	-5.6	1.059	0	No
RDW-SD^Z	-7.3	-0.6	1.3	0	No
Uric acid^z	-4.1	-0.4	1.211	0	No

Abbreviations: Alpha-hydroxybutyrate dehydrogenase; INR, International normalized ratio; LVFS, Left ventricular fractional shortening;

LVEF, Left ventricular ejection fraction; RDW-SD, Red blood cell distribution width-SD; VIF, variance inflation factors; "Z", Z-Score normalization.

Note: After removing the collinearity factors, it was finally found that when WBC(White blood cell count), LD1(Lactic dehydrogenase-1), Na^+(Serum sodium), Cl^-(Serum chlorine), PT(Prothrombin time), ALB(Albumin) were included or excluded from the equation, the initial regression coefficient changed by more than 10%, so these factors were included in the model for adjustment. To prevent non-linear relationships from affecting the model, we grouped these factors according to tertiles and treated them as categorical variables before adjustment.

Supplementary Figure 1. Flow chart showing the process of patient recruitment for the study population.

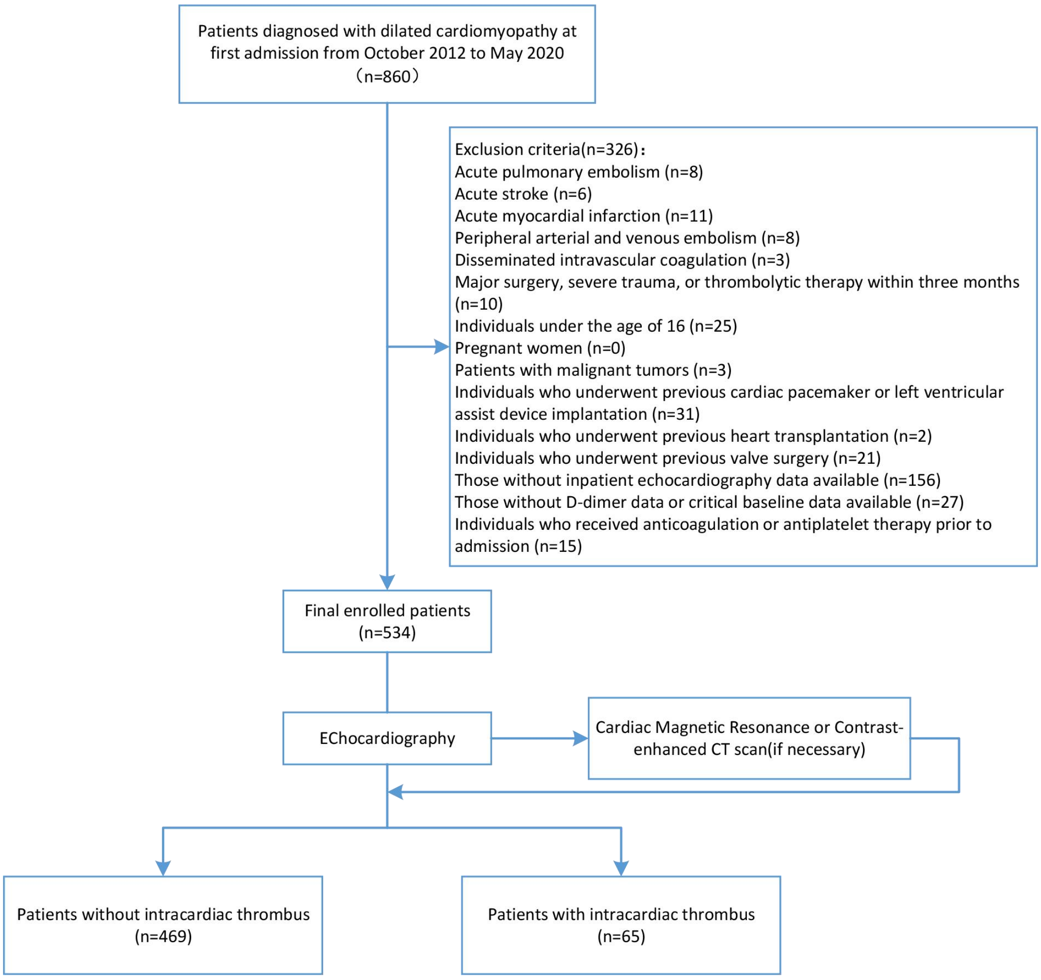

Supplement: Supplementary file 1 — Supplementary Information. [file 41598_2023_45077_MOESM1_ESM.pdf]
